# Native valve dual pathogen endocarditis caused by *Burkholderia cepacia* and *Aspergillus flavus* – a case report

**DOI:** 10.1099/jmmcr.0.005143

**Published:** 2018-03-08

**Authors:** Nargis Sabir, Aamer Ikram, Adeel Gardezi, Gohar Zaman, Luqman Satti, Abeera Ahmed, Tahir Khadim

**Affiliations:** ^1^​ Armed Forces Institute of Pathology, National University of Medical Sciences, Islamabad, Pakistan; ^2^​ National Institute of Health, Islamabad, Pakistan

**Keywords:** native valve endocarditis, dual infection, polymicrobial endocarditis, *Burkholderia cepacia*, *Aspergillus flavus*, MICs

## Abstract

**Introduction:**

Infective endocarditis (IE) is an important clinical condition with significant morbidity and mortality among the affected population. A single etiological agent is identifiable in more than 90 % of the cases, however, polymicrobial endocarditis (PE) is a rare find, with a poor clinical outcome. Here we report a case of native valve dual pathogen endocarditis caused by *Burkholderia cepacia* and *Aspergillus flavus* in an immunocompetent individual. It is among unique occurrences of simultaneous bacterial and fungal etiology in IE.

**Case presentation:**

A 30-year-old male was admitted to a cardiology institute with complaints of low grade intermittent fever and progressive shortness of breath for last two months. He was a known case of rheumatic heart disease and had suffered an episode of IE three years ago. On the basis of clinical presentation and the results of radiological investigations, a diagnosis of infective endocarditis was made. Paired blood samples for culture and sensitivity, sampled before the commencement of antimicrobial therapy, yielded growth of *Burkholderia cepacia* which was highly drug resistant. Sensitivity results-directed therapy consisting of tablet Trimethoprim–Sulfamethoxazole, two double-strength tablets 12 hourly, and Meropenem, 1 g IV every 8 h, was commenced. Despite mild relief of fever intensity, overall clinical condition did not improve and double valve replacement therapy was carried out. Excised valves were sent for microbiological analysis. *Burkholderia cepacia* was grown on tissue culture with a similar antibiogram to that previously reported from the blood culture of this patient. Direct microscopy of section of valvular tissue with 10 % KOH revealed abundant fungal hyphae. Patient serum galactomannan antigen assay was also positive. Histopathological examination of vegetations also revealed hyphae typical of species of the genus *Aspergillus*. The patient was successfully treated with meropenem, trimethoprim–sulfamethoxazole and voriconazole.

**Conclusion:**

The hallmark of successful treatment in this case was exact identification of pathogens, antibiogram-directed therapy and good liaison between laboratory experts and treating clinicians.

## Introduction

Infective endocarditis (IE) is an important clinical condition with significant morbidity and mortality among the affected population. A single etiological agent is identifiable in more than 90 % of the cases, [1] however, polymicrobial endocarditis (PE) is a rare find, with poor clinical outcome [2].

*Burkholderia cepacia* is an aerobic, Gram-negative rod [3], mostly a nosocomial pathogen that particularly infects patients with cystic fibrosis and chronic granulomatous disease [4]. It is also implicated in infective endocarditis among intravenous drug users and in patients with prosthetic valve replacement [5].

Fungal IE is a rare occurrence. Members of the genus *Aspergillus* are a known cause of invasive tissue infections with higher risk of infection in specific population to include pre-existing valvulopathy, parenteral nutrition, immunosuppression, broad-spectrum antibiotic regimens and intravenous drug abuse [6]. Valvular infection by this fungus is uncommon but carries a poor prognosis and high mortality rate [7].

Here we report a case of native valve dual pathogen endocarditis caused by *Burkholderia cepacia* and *Aspergillus flavus* in an immunocompetent individual. It is among unique occurrences of simultaneous bacterial and fungal etiology in IE.

## Case report

A 30-year-old male was admitted to a cardiology institute with complaints of low grade intermittent fever and progressive shortness of breath for the previous two months. He was a known case of rheumatic heart disease and had suffered an episode of IE three years ago. He was a non-smoker with no history of intravenous drug abuse and was linked to a construction business but presently unemployed due to failing health. On general physical examination, the patient had low grade fever (99 °F), tachycardia (110 beats min^−1^), hypotension (90/60 mm Hg), mild pallor and bilateral pitting ankle edema.

Chest auscultation revealed displaced apex beat and pan- systolic murmur radiating to axilla with bilateral basal crepitation. Massive cardiomegaly with interstitial edema and bilateral mild pleural effusion was seen on plain chest X-ray. 2D-Echocardiography revealed a dilated left ventricle, severe aortic and mitral regurgitation, with ejection fraction reduced to 30 %. Trans-esophageal echocardiography showed a deformed calcified mitral valve and large 14×17 mm vegetation on the aortic valve, dilated left ventricle and grade 3 mitral and aortic regurgitation. With mild anaemia and normal biochemical profile on lab testing, his generalized condition started deteriorating over the next two days with high grade fever, neutrophil leukocytosis (15.5×10^9^ l^−1^) and markedly raised C-reactive protein (96 mg dl^−1^). Empirical antimicrobial treatment consisting of intravenous Ceftriaxone 2 g IV once daily, Gentamicin 60 mg IV 8 hourly and Vancomycin 120 mg 8 hourly (target trough concentration of 15–20 µg ml^−1^) was initiated.

## Diagnosis

On the basis of clinical presentation and the results of radiological investigations, a diagnosis of infective endocarditis was made. Paired blood samples (from both arms to increase yield and to rule out contamination) were sent for culture and sensitivity testing before the commencement of antimicrobial therapy and yielded growth of *Burkholderia cepacia* which was highly drug resistant. Antimicrobial susceptibility of isolates was tested using a semi-automated continuous monitoring system VITEK-2 (bioMérieux). Both the isolates from paired blood cultures had similar antibiograms. Minimum Inhibitory Concentrations (MICs) breakpoints showed the isolates to be highly drug resistant and sensitive only to Trimethoprim–Sulfamethoxazole and Meropenem (Table 1). Sensitivity results directed therapy consisting of Trimethoprim–Sulfamethoxazole, two double-strength tablets 12 hourly and eropenem, 1 g IV every 8 h was commenced. Despite mild relief of fever intensity, overall clinical condition did not improve and double valve replacement therapy was carried out.

**Table 1.** Antibiotic susceptibility of *Burkholderia cepacia*

**Antibiotics**	**Disk zone in mm**	**MIC’s Breakpoints µg ml^−1^**	**Interpretation according to CLSI Break points**
Ceftazidime	15	34	Resistant
Meropenem	22	2	Sensitive
Minocycline	12	18	Resistant
Levofloxacin		16	Resistant
Trimethoprim–sulfamethoxazole	18	<2/38	Sensitive

Excised valves were sent for microbiological analysis. *Burkholderia cepacia* was grown on tissue culture with a similar antibiogram to that previously reported from the blood culture of this patient. Direct microscopy of sections of valvular tissue with 10 % KOH revealed abundant fungal hyphae. Valves tissue fungal culture and sensitivity yielded bright yellow to green, floccose to granular growth of fungus with a creamy reverse after seven days of incubation. On microscopic examination the fungus showed vesiculate conidiophores with numerous large round to elliptical smooth double-walled conidia. On the basis of colony morphology and microscopic findings, the fungus was recognized as *Aspergillus flavus*. The MIC of the isolated fungus was determined by the broth microdilution method. The isolate was found to be sensitive only to Voriconazole and resistant to Amphotericin B, Itraconazole and Fluconazole.

To support the diagnosis of fungal etiology, serum galactomannan assay along with histopathology of the excised tissue was also carried out. Positive serum galactomannan assay confirmed the invasive trend of the grown fungus. Using periodic acid–Schiff (PAS) and Grocott–Gomori’s methenamine silver stain, small and uniform septate hyphae with dichotomous branching hyphae resembling species of the genus As*pergillus* were seen in the excised tissue and the environmental contamination of the excised valves specimen.

## Treatment

There was a definite improvement in the rapidly deteriorating condition of the patient after the valvular excision but the fever did not subside completely and occasional spikes still occurred with continued mild dyspnea. Treatment initially comprised of Trimethoprim–Sulfamethoxazole and Meropenem. With the evidence of fungal etiology playing its role in the disease process, IV Voriconazole, 400 mg 12 hourly for 2 doses then 200 mg every 12 h, was also added to the treatment regimen. The patient responded well to treatment.

## Outcome and follow-up

Due to the improved clinical response, the patient was discharged after two weeks on oral Voriconazole, 200 mg 12 hourly, and advised follow up. Two weeks after completing antifungal therapy, his serum galactomannan assay turned negative.

## Discussion

Polymicrobial endocarditis (PE) involving native valve is a rare occurrence. PE with bacterial etiology most frequently includes coagulase-negative *Staphylococci* (CoNS) in combination with *Enterococci*, Gram-negative bacilli or *Streptococcus viridans* [8, 9]. There are only few case reports of PE with bacterial and fungal etiology. Such reports include PE with *Streptococcus sanguis* and *Phialemonium aff. curvatum* [10], *Listeria monocytogenes* and *Acremonium* spp [11], *Burkholderia cepacia* and *Candida dubliniensis* [12] and PE due to *Candida tropicalis* with *Staphylococcus aureus* [13]. This is, to our knowledge, the first case of PE involving *Burkholderia cepacia* and *Aspergillus flavus.*

There are some known risk factors associated with PE. Intravenous drug abusers and/or patients with prosthetic valves have higher incidences of such infections. In addition, patients with compromised immune status, as seen in malignancy, organ transplantation and those on chemotherapy or on broad-spectrum antibiotics, or patients with co-morbidities (like diabetes mellitus, chronic renal failure, chronic granulomatous diseases and cystic fibrosis) [14] are also at significant risk of developing PE. Our patient was not an intravenous drug user and had a native valve infection. Nonetheless, he had bilateral valvulopathy and a history of rheumatic heart disease. This valvular damage was complicated by the fact of involvement of both the atrial valves. This particular feature may have increased the propensity for development of PE.

Dual infections pose a diagnostic inconvenience, especially when pathogens are recoverable from different specimens. We had the evidence of bacterial etiology on the basis of the isolation of *Burkholderia cepacia* from paired blood cultures. We never expected any fungal etiology or PE in our patient in the first place. Growth of a species of the genus *Aspergillus* from the excised valve tissue led us to the fungal etiology. Blood cultures are almost always negative in *Aspergillus* infections. Diagnosis of invasive aspergillosis requires histopathological evidence of tissue invasion by small and uniform, dichotomous, hyphae with septate branching at regular intervals and/or culture findings positive for *Aspergillus* spp. from tissue [15]. There could have been a chance of post-operative contamination of valves by ubiquitous *Aspergillus flavus* posing a challenge to our diagnosis. A serological marker of invasive fungal infection, i.e. serum galactomannan, was also looked for and turned out to be positive. The high specificity of serum galactomannan for invasive aspergillosis [16] along with histopathological evidence of *Aspergillus* in the tissue confirmed the presence of *Aspergillus flavus* as a pathogen and not merely a contaminant.

The occurrence of dual infections is compatible with one agent predisposing for another. The sequence of events is often difficult to determine, but we propose that severe damage to atrial valves by rheumatic heart disease contributed to providing the nidus for *Aspergillus* infection. Following occupational exposure to *Aspergillus flavus,* the fungus-induced endothelial damage could have favoured bacterial infection by *Burkholderia cepacia*, which is cosmopolitan and present in the hospital environment.

Treatment of PE requires combined medical and surgical standards of care [13]. We were able to achieve some degree of clinical response in the light of blood culture and sensitivity but significant valvular damage had made surgical intervention inevitable. Surgical removal of valves made a great difference to the clinical condition but still we were not able to achieve the best response anticipated. Probably some of the fungal remnants in the tissues due to the invasive nature of the fungus had been causing their effects. Addition of Voriconazole was a valuable step that has resulted in achieving the desired clinical outcome.

The hallmark of successful treatment in this case was exact identification of the pathogens, antibiogram-directed therapy and good liaison between laboratory experts and treating clinicians.
